# Thermodynamic Evaluation of the Interactions between Anticancer Pt(II) Complexes and Model Proteins

**DOI:** 10.3390/molecules26082376

**Published:** 2021-04-19

**Authors:** Chiara Pelosi, Francesca Saitta, Caterina Zerino, Giovanni Canil, Tarita Biver, Alessandro Pratesi, Celia Duce, Dimitrios Fessas, Chiara Gabbiani, Maria Rosaria Tiné

**Affiliations:** 1 Department of Chemistry and Industrial Chemistry, University of Pisa, Via G. Moruzzi 13, 56124 Pisa, Italy; chiara.pelosi@dcci.unipi.it (C.P.); zerinocaterina@gmail.com (C.Z.); giocanil@gmail.com (G.C.); tarita.biver@unipi.it (T.B.); alessandro.pratesi@unipi.it (A.P.); celia.duce@unipi.it (C.D.); 2 Dipartimento di Scienze per gli Alimenti, la Nutrizione e l’Ambiente, DeFENS, Università degli Studi di Milano, Via Celoria 2, 20133 Milano, Italy; francesca.saitta@unimi.it (F.S.); dimitrios.fessas@unimi.it (D.F.); 3 Department of Pharmacy, University of Pisa, Via Bonanno Pisano 6, 56126 Pisa, Italy

**Keywords:** antitumoral complex, Pt(II) coordination, calorimetry, binding mechanism, interaction complex-protein

## Abstract

In this work, we have analysed the binding of the Pt(II) complexes ([PtCl(4′-phenyl-2,2′:6′,2″-terpyridine)](CF_3_SO_3_) (**1**), [PtI(4′-phenyl-2,2′:6′,2″-terpyridine)](CF_3_SO_3_) (**2**) and [PtCl(1,3-di(2-pyridyl)benzene) (**3**)] with selected model proteins (hen egg-white lysozyme, HEWL, and ribonuclease A, RNase A). Platinum coordination compounds are intensively studied to develop improved anticancer agents. In this regard, a critical issue is the possible role of Pt-protein interactions in their mechanisms of action. Multiple techniques such as differential scanning calorimetry (DSC), electrospray ionization mass spectrometry (ESI-MS) and UV-Vis absorbance titrations were used to enlighten the details of the binding to the different biosubstrates. On the one hand, it may be concluded that the affinity of **3** for the proteins is low. On the other hand, **1** and **2** strongly bind them, but with major binding mode differences when switching from HEWL to RNase A. Both **1** and **2** bind to HEWL with a non-specific (DSC) and non-covalent (ESI-MS) binding mode, dominated by a 1:1 binding stoichiometry (UV-Vis). ESI-MS data indicate a protein-driven chloride loss that does not convert into a covalent bond, likely due to the unfavourable complexes’ geometries and steric hindrance. This result, together with the significant changes of the absorbance profiles of the complex upon interaction, suggest an electrostatic binding mode supported by some stacking interaction of the aromatic ligand. Very differently, in the case of RNase A, slow formation of covalent adducts occurs (DSC, ESI-MS). The reactivity is higher for the iodo-compound **2**, in agreement with iodine lability higher than chlorine.

## 1. Introduction

Since the discovery of its therapeutic power in 1965, cisplatin has become the chemotherapeutic agent of choice for the treatment of different types of cancers, e.g., testicular, ovarian and bladder cancer, melanoma, non-small cell lung cancer (NSCLC), small cell lung cancer (SCLC), lymphomas and myelomas [1,2]. Despite its wide use, cisplatin presents several side effects (neurotoxicity, nephrotoxicity, myelosuppression and ototoxicity); these are only partially reversible when the treatment is interrupted [3,4]. The accumulation of metal ions in the body after long-term or high-dose therapy was found [5]. Besides, some types of cancer are intrinsically insensitive to treatment with cisplatin (innate resistance), while other tumours develop resistance only during chemotherapy (acquired resistance) [3].

In the past, the scientific community working on anticancer metal compounds has mainly focused on interactions with DNA, the commonly accepted primary target for platinum compounds [6]. Subsequently other studies proved that only 5–10% of covalently bound cell-associated cisplatin is found in the DNA fraction, whereas 75–85% of the drug binds to proteins [7]. This finding opens the way to move the accepted paradigm on the mechanism of action of Pt-based compounds to an enlarged paradigm involving protein metalation [8]. The attention was thus also directed to the understanding of metallodrug-protein interactions. These are crucial, not only concerning apoptosis but also for unwanted side-effects (due to indiscriminate protein binding), drug resistance and even possibly drug delivery and storage [9,10].

Nowadays, cisplatin and related compounds are known to bind to several classes of proteins with different roles, including transporters, antioxidants, electron transfer proteins, DNA-repair proteins, as well as proteins/peptides used as model systems to characterize the reactivity of metallodrugs in vitro, but that are also present in vivo [11]. In this regard, studies on the interactions of cisplatin and its analogues with proteins are fundamental in determining the overall pharmacological and toxicological profile of platinum drugs [11]. After the intravenous administration to the patient, most of the platinum deriving from cisplatin (from 65 to 90%) forms a binding with many plasma proteins (e.g., serum albumin, haemoglobin and transferrin) [12], due to the strong affinity of platinum towards thiol groups present in amino acids, such as cysteine residues [13]. Other proteins with which cisplatin could interact during its life are copper transport proteins (Ctr1 and Ctr2) and ATPases, involved in the cisplatin cellular uptake, DNA damage recognition proteins, that limits its cytotoxic action, and many others in smaller percentages [12].

The consensus on the crucial role of the binding of metallodrugs with protein targets has grown [14,15]. Some of us were already involved in the study of metallodrugs interaction with small molecular models mimicking the protein coordinative sites [16,17] and with proteins of bigger size [18,19,20].

**Scheme 1:**
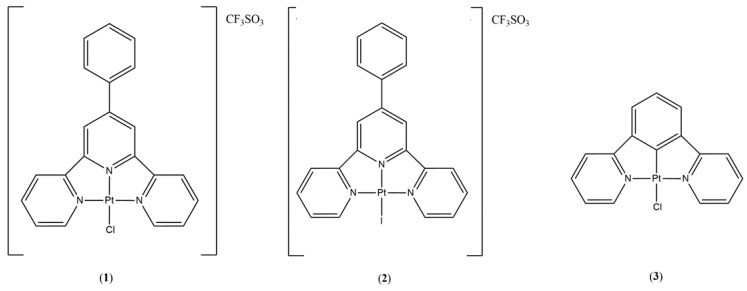
Platinum (II) complexes studied: [PtCl(4′-phenyl-2,2′:6′,2″-terpyridine)](CF_3_SO_3_) (**1**), [PtI(4′-phenyl-2,2′:6′,2″-terpyridine)](CF_3_SO_3_) (**2**) and [PtCl(1,3-di(2-pyridyl)benzene)] (**3**).

In this context, we evaluated the interactions and relevant thermodynamic parameters for the interaction of three Pt(II) anticancer complexes, potential cisplatin substitutes, with selected model proteins, namely hen egg-white lysozyme (HEWL) and ribonuclease A (RNase A). We focused on the positively charged complexes [PtCl(4′-phenyl-2,2′:6′,2″-terpyridine)](CF_3_SO_3_) ([PtCl(phterpy)](CF_3_SO_3_)); (**1**) and [PtI(4′-phenyl-2,2′:6′,2″-terpyridine)](CF_3_SO_3_) ([PtI(phterpy)](CF_3_SO_3_); (**2**) (Scheme 1), as they showed promising features in previous studies [21,22]. We added to the study the neutral complex [PtCl(1,3-di(2-pyridyl)benzene)] ([PtCl(DPB)]); (**3**) as a reference to evaluate reactivity differences due to the absence of the charge in the metal centre and of a less extended ligand. Besides, samples **1** and **2** are Pt(II)-coordination compounds with an NNN-type ligand, while the third complex is an organometallic compound with an NCN-type ligand. The 4′-phenyl-2,2′:6′,2″-terpyridine ligand has been already investigated by some of us as a stabile tridentate conjugate ligand for the Pt(IV) centre and, behaving like an antenna, gathers the light to promote the photoreduction to the corresponding Pt(II) analogue [21,23]. Despite these Pt(II) compounds did not show any intercalative properties towards the DNA, they turned out to be very cytotoxic in vitro against the A2780 ovarian cancer cell line [21]. These preliminary results spurred us to investigate further the reactivity of this class of compounds towards model proteins.

The study of the possible interactions with proteins was performed with complementary techniques, i.e., differential scanning calorimetry (DSC), UV-Vis spectroscopy and mass spectrometry. Calorimetry is a powerful tool for detecting the perturbations on the protein unfolding process caused by an external agent, e.g., polymers or drugs [24,25,26,27]. UV-Vis titrations and, in general, spectroscopic studies (absorbance and fluorescence) are approaches that may appear simple but are, instead, tricky and powerful procedures that give interesting information on the binding mechanism [28,29]. Mass spectrometry gave additional value to the study and enabled to define the molecular identity of the adduct formed with the binding.

Overall, we enlightened the presence of a peculiar binding mode for the two different proteins and highly dependent on the electronic and steric properties given by the ligand to the metallic centre.

## 2. Results and Discussion

### 2.1. Stability over Time of Metal Complex in Solution

The stability of the complexes **1**–**3** in the experimental conditions (0.1 M acetate buffer at pH = 4.5) was evaluated by UV-Vis spectrophotometry. Samples **1** and **2** were stable in the buffer for at least 24 h, while sample **3** presented variations occurring over time (Figures S1 and S2 in the Supplementary Materials).

The different reactivity of the three complexes was probably due to electronic factors. The phenyl and pyridine rings behave as π-acceptors with low steric hindrance, increasing the electrophilicity of the metallic centre. The higher lability of complex **3** was probably cuased by the presence, in a *trans* position with respect to the chloride, of a phenyl carbon that is a strong σ-donor, that labilizes the Pt-Cl bond and cause a high destabilization of the ground state with a consequent increase in intrinsic reactivity. Note that no such a significant band shape change occurs in pure water, except for a slight absorbance reduction in the UV region (Figure S2d in the Supplementary Materials). Therefore, we may suppose that, in the buffer, an exchange between the chloride and an acetate group could take place, changing the coordination sphere around the metal centre. This hypothesis was supported by the observance of a change in the solution colour in the buffer, going from yellow to blue over time and with the correspondent born of an intense new band peaked at 578 nm (Figure S2a–c in the Supplementary Materials). The UV-vis spectra reached equilibrium after about 40 h, confirming the stability of the new complex formed.

### 2.2. Protein Binding by Differential Scanning Calorimetry

The interaction between the complexes **1**–**3** and the model proteins HEWL and RNase A was evaluated by differential scanning calorimetry (DSC). Table 1 gathers the relevant thermodynamic parameters obtained at different incubation times.

**Figure 1:**
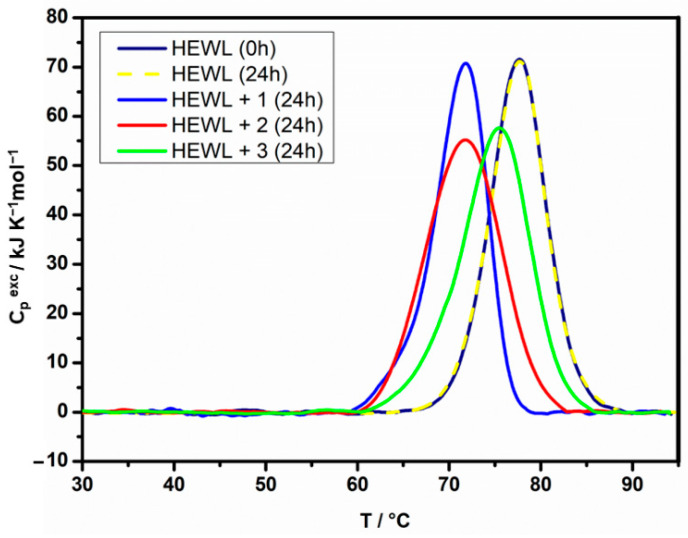
Micro-DSC thermograms obtained for HEWL alone and in the presence of compounds **1**–**3** (in a molar ratio protein:complex 1:5) in acetate buffer (0.1M, pH 4.5), after 24 h of incubation at 37.0 °C. The thermogram obtained for HEWL without incubation (0 h) is also reported for sake of comparison.

The effect of HEWL interaction with the three complexes was investigated after 24 h and 48 h of incubation. Figure 1 reports the comparison of the free HEWL and HEWL + complex systems after 24 h of incubation.

We observe that the interactions with all complexes lead to a thermodynamic protein destabilization, mainly in terms of entropic contribution. Its magnitude depends on the chemical nature of the compound, whilst no significant variation in the Δ_d_*H*° are observed (Table 1). In particular, both **1** and **2** lead to a similar downshift of the protein denaturation temperature (*T*_d_), even though the peak obtained by the interaction with compound **2** is wider, index of less cooperative process. Instead, the protein exhibits a minor decrease in the *T*_d_ caused by the interaction with complex **3**. The same picture was obtained after 48 h of incubation (Figure S4 in the Supplementary Materials), indicating that the interaction processes have already reached saturation and no further kinetic phenomena will occur. Also, no aggregation effects were observed. According to the literature [30,31,32], thermodynamic exploitation may be carried out in these conditions. However, despite the denaturation process of the free HEWL is well fitted by a single-step thermodynamic equilibrium model (Figure S3a in the Supplementary Materials), the fit attempts performed on the different HEWL + complex systems with various thermodynamic models failed [32,33,34]. In other words, the action of the complexes on the protein thermal stability cannot be described as a simple equilibrium binding processes. This missed thermodynamic description, together with the asymmetries of the thermograms exhibited by the HEWL + complex systems, is compatible with a scenario for which the protein interaction with the compounds is non-specific. Hydrophobic interactions between protein’s and complexes’ aromatic rings or electrostatic interactions in the case of the charged complexes **1** and **2** may be at play and produce statistical distributions of several populations in which the protein differently coordinates the metal complexes.

**Figure 2:**
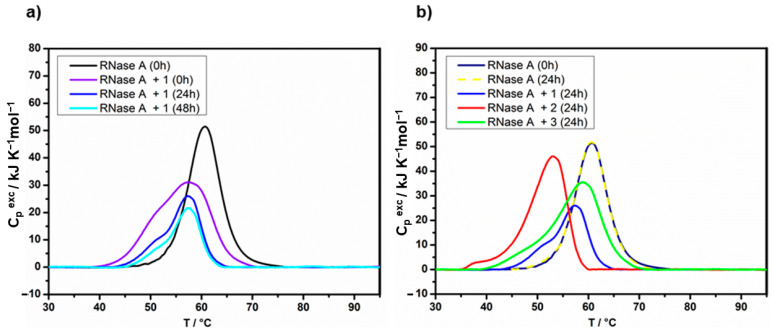
(**a**) Nano-DSC thermograms obtained for RNase A alone or in the presence of sample **1** after 0, 24 and 48 h of incubation at 37 °C in 0.1 M acetate buffer at pH = 4.5; (**b**) Nano-DSC thermograms for RNase A alone or in the presence of the samples **1**–**3** (protein:complex 1:5 molar ratio) after 24 h of incubation at 37.0 °C in 0.1 M acetate buffer at pH = 4.5.

Besides, we investigated the interaction of the three complexes with RNase A (Figure 2). We studied the effects of the binding on the protein denaturation process after 24 h and 48 h of incubation. Although the RNase A denaturation peak can be fitted through a single-step thermodynamic equilibrium model (Figure S3b in the Supplementary Materials), metastable states and kinetic effects were observed in the RNase A + complex systems.

Figure 2a reports the evolution of the thermograms observed for RNase A + **1** at different incubation times, as an example. We note a destabilizing action that shifts the thermograms towards lower temperatures. Furthermore, the thermograms become shouldered, and despite the profiles remain almost similar, the overall enthalpy progressively decreases, highlighting that the protein irreversibly denatures during the incubation time. Similar kinetic effects are also observed for compounds **2** and **3**, even though with lower rates. A deeper kinetic study about such irreversible phenomena is beyond the scope of this paper. Instead, to compare the action of the complexes, we limit our description to the intensive properties, i.e., the temperature range of denaturation and the thermogram profiles observed.

For the sake of homogeneity with the profiles reported for HEWL, Figure 2b shows a comparison of the three systems after 24 h of incubation at 37 °C, whereas the respective relevant thermodynamic parameters are reported in Table 1. We observe that compounds **2** and **3** behave similarly to the HEWL’s analogues (see Figure 1), whereas a minor destabilizing effect (if we exclude the irreversible time-depending phenomena) was produced by **1**. Furthermore, asymmetries and shoulders are evident in all cases, strengthening the scenario of non-specific interactions leading to a distribution of multiple protein-complex species.

### 2.3. Protein Binding by UV-Vis Titrations

**Figure 3:**
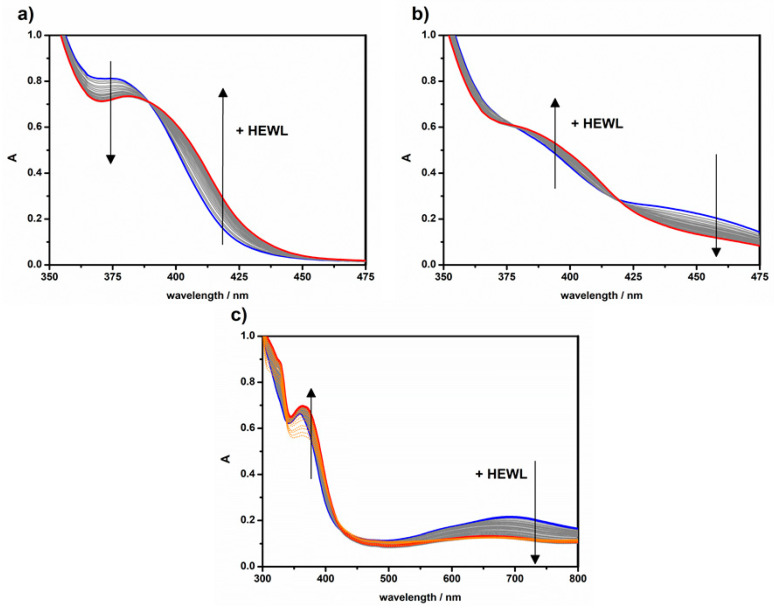
Absorption spectra of the platinum complexes in the presence of increasing amounts of the HEWL protein (from blue to red). (**a**) Complex **1**, C_PtL_ = 1.30 × 10^−4^ M; C_HEWL_ from 0 to 3.57 × 10^−4^ M, (**b**) complex **2**, C_PtL_ = 1.30 × 10^−4^ M; C_HEWL_ from 0 to 4.59 × 10^−4^ M, (**c**) complex **3**, C_PtL_ = 1.71 × 10^−4^ M; C_HEWL_ from 0 to 5.44 × 10^−4^ M (but precipitation occurs above C_HEWL_ > 4 × 10^−4^ M, these spectra are reported as dotted lines); acetate buffer 0.1 M, pH = 4.5, 25 °C.

The changes in the UV-Vis spectra of the complex under increasing protein content enlighten the occurrence of a binding reaction. Figure 3 shows the result of a spectrophotometric titration of the complex **1** with HEWL, which confirms the presence of an interaction taking place between **1** and the biosubstrate. Complex **1** showed a characteristic absorption maximum at 378 nm, which was red-shifted by about 3 nm and underwent hypochromism. The presence of a well-defined isosbestic point at 390 nm suggested a relatively simple equilibrium (one dominating type of bound species).

The interaction between the platinum metal complex **1** and HEWL may be in a first approximation represented by the following reaction (Equation(1)):

HEWL + PtL ⇄ HEWL-PtL,(1)

where HEWL is the protein, PtL the unbound Pt(II)-complex and HEWL-PtL the resulting adduct. Under these circumstances, the analysis of spectrophotometric titrations can be performed using Equation (2):

(2) $$\frac{C_{\mathrm{PtL}}C_{\mathrm{HEWL}}}{\Delta A}+\frac{\Delta A}{\Delta \varepsilon^{2}}=\left(C_{\mathrm{PtL}}+C_{\mathrm{HEWL}}\right)\frac{1}{\Delta \varepsilon }+\frac{1}{K\Delta \varepsilon }$$

Here C_PtL_ and C_HEWL_ are the total analytical concentrations of respectively complex **1** and HEWL, ΔA = A − ε_PtL_C_PtL_ is the absorbance signal change, Δε = ε_HEWL_-_PtL_ − ε_PtL_ is the amplitude of the binding isotherm (Figure S5a from Supplementary Materials) and K is the equilibrium constant associated to equilibrium (1). As Δε value is not known, an iterative procedure is needed that first disregards the ΔA/Δε^2^ term and then calculates it iteratively by reciprocal of the slope of the obtained straight line [35]. At convergence, the value of K is calculated by the slope/intercept ratio of the straight-line interpolating data points (Figure S5b from Supplementary Materials). Table 2 collects the K values obtained.

The apparent interaction constant can also be obtained using Equation (3), which is an alternative form of the Scatchard equation [28]:

(3) $$\frac{C_{\mathrm{HEWL}}\left(C_{\mathrm{PtL}}\Delta \varepsilon -\Delta A\right)}{\Delta A}=\frac{1}{\mathrm{nK}}+\frac{\left(C_{\mathrm{PtL}}\Delta \varepsilon -\Delta A\right)}{\Delta \varepsilon }\frac{1}{n}.$$

Here, n is the number of equivalent sites per protein unit and Δε value comes from the binding isotherm and is confirmed by the analysis according to Equation (2). An example of data analysis is provided in the Supplementary Materials (Figure S5c); results are in Table 2. The number of sites per unit of protein is close to one, confirming the suitability of Equation (2) to treat the data.

The same procedure was applied to complex **2**. Examples of the spectral variation observed are shown in Figure 3b, and data treatment is reported in the Supplementary Materials as Figure S6. When increasing amounts of HEWL are added, the absorption band of **2** at ca. 450 nm fades out and a new band appears in the 380–410 nm range. An isosbestic point at 420 nm suggests the presence of simple equilibria. The spectrophotometric data were analysed according to Equations (2) and (3), and the results are shown in Table 2.

Complex **3**, unlike the previous two, showed precipitation for C_HEWL_/C_PtL_ > 2.5. As shown in Figure 3c, by increasing HEWL concentration, the absorption band at 360 nm is slightly red-shifted by about 5 nm and shows hyperchromism, while that at 700 nm shows a marked hypochromism and blue-shifts while fading. The precipitation process lowered the robustness of the binding models and concurrently the precision of the binding parameters; no K evaluation was possible, in particular by Equation (3) (Table 2 and Figure S7 in the Supplementary Materials). However, it can be concluded that for this species the affinity for the protein is much lower with respect to the other complexes. Note that, in the presence of HEWL, there is no born of the peak at 578 nm related to acetate coordination (and this should be visible in the time range of sample preparation + titration). Also, the final mixture was left in the spectrophotometer, but no substantial change of the spectrum occurred even on longer time ranges. It means that acetate coordination is suppressed in the presence of protein.

**Figure 4:**
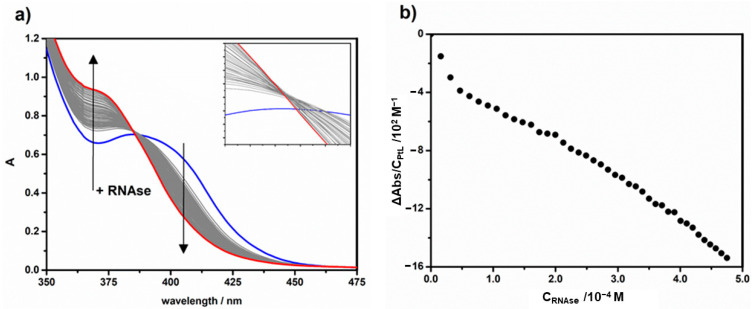
Absorption spectra of the platinum complex **1** in the presence of increasing amounts of the RNase A protein (from blue to red) (**a**) and relevant binding isotherm at 410 nm (**b**). C_PtL_ = 1.30 × 10^−4^ M; C_RNase_ from 0 to 4.75 × 10^−4^ M, inset is the amplification of the point where spectra are crossing; acetate buffer 0.1 M, pH = 4.5, 25 °C.

Turning now to RNase A interactions, the absorption spectra of **1** in the presence of increasing protein concentration are shown in Figure 4a. With increasing additions of RNase A, the absorption band of complex **1** is blue-shifted by about 10 nm, and its intensity is enhanced. Therefore, the effect is here opposite to what observed for HEWL. The binding isotherm (Figure 4b) corresponds to a complex trend which suggests the presence of multiple and cooperative equilibria (also in agreement with the non-perfect isosbestic point shown in the inset of Figure 4a), finally leading to some precipitation. Under these circumstances, no quantitative data analysis according to the equations above is possible.

As for **2**, when increasing amounts of proteins are added, the absorption band at 430 nm undergoes hypochromicity, and a new band is formed in the range between 350 and 380 nm (Figure S8a in the Supplemetary Materials). For **2**, application of Equations (2) and (3) yielded, respectively, K = (5 ± 2) × 10^3^ M^−1^, K′ = (3 ± 1) × 10^4^ M^−1^ and *n* = 0.5 (Figure S8c, d in the Supplementary Materials). Nevertheless, these values must be considered only indicative, since the binding isotherms are again significantly distorted (Figure S8b in the Supplementary Materials). This finding indicates that, also for complex **2**, multiple/cooperative equilibria with complex stoichiometries should be envisaged. In the case of **3**, the presence of precipitation effects inhibited any analysis of the binding process.

On the whole, as for the point of view of the UV-Vis titrations, we may conclude the following. First, the binding to HEWL occurs in a very different way to RNase A. A comparison between Figure 3a and Figure 4a supports this picture, as the spectrophotometric response of **1** upon binding is opposite in the case of the two biosubstrates. Note that UV-Vis titrations will reflect the events occurring on short time scales (1–2 h), whereas slow conformational changes of the protein and possible covalent binding will need longer times to take place. The complexes do bind to HEWL according to a simple equilibrium (in particular, no different binding types evidenced) that follows a 1:1 stoichiometry. The binding constants follow the order K(**1**) ≥ K(**2**) >> K(**3**). The significant spectral changes observed indicate that the aromatic part of the ligand is involved in the binding. For RNase A, besides precipitation phenomena taking place, the binding isotherms show complex trends suggesting a different and more complex binding mechanism.

### 2.4. Protein Binding by Mass Spectrometry

**Figure 5:**
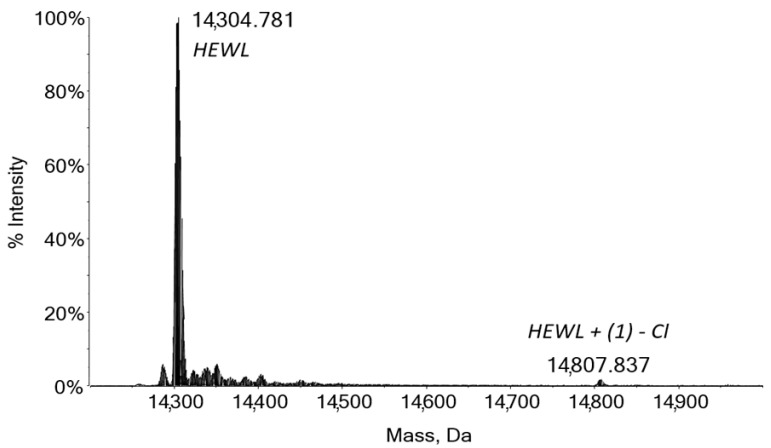
Deconvoluted ESI-Q-TOF mass spectrum of HEWL 10^−7^ M incubated for 24 h at 37 °C with **1** (1:3 protein/metal ratio) in ammonium acetate solution 2 × 10^−3^ M (pH 6.8).

The reactivity behaviour of complexes **1**–**3** towards the two model proteins was also evaluated using high-resolution ESI mass spectrometry. For these experiments, the proteins were treated in physiological-like conditions, using a well-established protocol [18,36,37] that can assure a protein reactivity as similar as possible to that of the physiological environment. Moreover, the spectra were acquired with a methodology able to preserve the protein in its native state, conserving unaltered all the covalent interactions formed in solution and most of the non-covalent ones [38]. From the inspection of the obtained spectra, clearly emerged two different reactivity patterns depending on the nature of the biomolecules. Specifically, in the case of HEWL, the main peak showed in the spectrum recorded after 24 h of incubation at 37 °C (14304 Da) belongs to the unreacted protein (Figure 5). Nevertheless, a very weak signal at 14,807 Da was also present and assignable to the adduct between HEWL and compound **1** without the chloride ligand. Interestingly, this signal was not conserved in the spectrum recorded after 48 h of incubation, and only the unreacted protein was present (Figure S9 from Supplementary Materials). This behaviour seems to be in accordance with a non-covalent interaction between the protein and the metal complex, probably giving an interaction mainly of electrostatic nature with the dicationic metal fragment and some negatively-charged amino acid residues, e.g., the aspartic acid.

Although the weak electrostatic interactions can be preserved in the gas phase during the ionization process [39,40], the spectrum recorded after 48 h of incubation with compound **1** did not show any adduct signal. This behaviour was probably due to the lability of this non-covalent adduct and its possible degradation in the measurement conditions. In the case of complex **2**, this behaviour was not evidenced even after 24 h of incubation (Figure S10 from Supplementary Materials). In this case, the presence of one iodide in place of one chloride could affect in a significant way the non-covalent interactions of this complex. Probably, being the iodide ligand of much bigger size with respect to the chloride and less prone to hydrolysis, complex **2** is not favoured, in the measurement conditions, to correctly approach the protein surface and establish the weak non-covalent interaction [41].

On the other hand, the neutral complex **3**, probably due to the absence of charge, did not show any electrostatic interaction with HEWL. The covalent interaction is also absent and its NCN coordinative environment confers to this complex a particular stabilisation to the metal centre, and thus, less reactivity (Figures S9 and S10 in the Supplementary Materials).

Contrarywise, when compound **2** reacted with RNase A, its reactivity behaviour was totally different. After the first 24 h of incubation, the spectrum showed two main signals at 13,682 and 13,780 Da corresponding to the unreacted RNase A and its adducts with the isobaric sulphate or phosphate anions, respectively (Figure S11 in the Supplementary Materials). From the literature, it was well established that the solution media used during the extraction and purification process of the commercially available RNase A can contain a small amount of phosphoric acid or sulphuric acid that is responsible for the formation of isobaric and undesired adducts with RNase A (+98 *m*/*z*) [42]. These adducts remain visible even when the protein is dissolved in a different medium, like here, where ammonium acetate solution has been employed. Beyond the unreacted protein, the spectra also showed two peaks corresponding to the metal adduct of these two protein forms at 14,184 and 14,283 Da. In this adduct, the reactive fragment corresponds to compound **2** without the iodide ligand.

**Figure 6:**
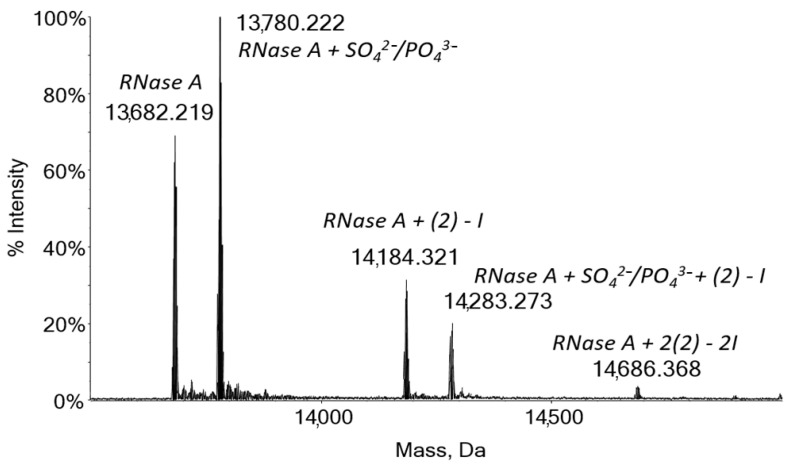
Deconvoluted ESI-Q-TOF mass spectrum of RNase A 10^−7^ M incubated for 48 h at 37 °C with **2** (1:3 protein/metal ratio) in ammonium acetate solution 2 × 10^−3^ M (pH 6.8).

The ESI mass spectrum obtained for RNase A after 48 h of incubation with complex **2** is reported in Figure 6. It shows that the two peaks at 14,184 and 14,283 Da (corresponding to the mono adduct of RNase A and its form with SO_4_^2−^/PO_4_^3−^) are still present, and their intensities appear to be remarkably increased with respect to 24 h. This is the typical behaviour of a covalent adduct with slow kinetics of the reaction, that proceeds during all the incubation period. As a further insight in this direction, note the appearance of a new small peak at 14,686 Da, corresponding to the bis adduct with the protein. The main peaks at 13,682 and 13,780 Da due to the unreacted protein forms are still present.

Similarly to compound **2**, complex **1** produced a protein adduct with a related intense signal at 14,183 Da (and 14,282 for the protein with SO_4_^2−^/PO_4_^3−^). This peak corresponds, also in this case, to the monoadduct of complex **1** with the loss of the chloride ligand. Surprisingly, after 48 h of incubation, the resulting spectra did not show any adduct signals (Figures S11 and S12 in the Supplementary Materials). Also, the intensity of the unreacted protein signal appears to be sensibly diminishing. In fact, from the data and experimental observations gathered with the other techniques described above, this is in accordance with an extensive adducts precipitation from the solution.

This protein aggregation phenomenon is evident with compound **1** and is absent with compound **2**. The real explanation of this different behaviour cannot be furnished at this stage of the study; however, since the reactive fragment is the same in both cases—i.e., Pt(4′-phenyl-2,2′:6′,2″-terpyridine)- the nature of the labile ligand released in solution could play a fundamental role in the protein aggregation.

Once again, complex **3** appears to be completely unreactive, also with the RNase A, and both its spectra recorded after 24 and 48 h of incubation displayed only the signals belonging to the unreacted protein (Figures S11 and S12 in the Supplementary Materials).

In conclusion, with the ESI-MS we gained further insight into the peculiar reactivity for these three platinum-based complexes confirming and deepening the experimental evidence obtained with DSC and UV-Vis spectrophotometry. Complex **1** forms with HEWL a small percentage of a non-covalent adduct that can be reasonably described as electrostatic. Probably due to the chemico-physical properties of the labile ligand, complex **2** is not able to establish the necessary electrostatic interaction with the protein to be revealed in MS as a non-covalent adduct. Taking into account the nephelauxetic effect of the ligands, the iodide can establish interaction with the Pt centre of more covalent nature with respect to the chloride, leading to a more stable complex where the labile ligand is more easily retained, and to a less propensity to give adducts of electrostatic nature.

On the contrary, both complexes **1** and **2** are reactive towards RNase A, giving a well-defined monoadduct and, in the case of complex **2**, a bis-adduct. Lastly, the neutral compound **3** is unreactive with both proteins, and in its MS spectra, there are no indications for the formation of covalent adducts nor electrostatic ones.

Overall, in agreement with the other techniques, ESI-MS showed a stronger affinity of the complexes for RNase A with respect to HEWL. Looking more in detail at the protein’s structure, it is known from the literature that both HEWL and RNase A possess some amino acid side chains with which platinum could interact covalently [43,44,45]. The differential reactivity found in this work could be tentatively explained by keeping in mind the molecular geometry of the reactive fragment. The three-coordinated platinum centre is surrounded by the terpyridine ligand, endowed with three aromatic rings that confer rigidity to this planar structure. Moreover, the Pt atom has only one free coordinative site requiring the right spatial orienteering of the molecule to establish a covalent bond. Besides, the spatial hindrance of the terpyridine ligand could not allow the interaction with the protein amino acid side chains that are not fully solvent-exposed, as it probably occurs in the case of HEWL.

## 3. Materials and Methods

### 3.1. Materials

The metal complexes **1**–**3** were synthesized as described in the cited papers [21,22,46]. Hen egg-white lysozyme (HEWL) and ribonuclease A from bovine pancreas (RNase A) were purchased from Sigma-Aldrich (St. Louis, MO, USA) and used without further purification or manipulation. LC-MS grade water, sodium acetate, acetic acid and dimethylsulfoxide (DMSO) were also purchased from Sigma-Aldrich. When not otherwise specified, all reagents were analytical grade purity and were used without further purification.

Stock solutions of the metal complexes (1.0 mM) were prepared in DMSO by weight and kept in the fridge; working solutions were obtained by diluting the stocks with the desired buffer. The exact protein concentration in buffer was calculated by UV-Vis absorbance spectrophotometry, using ε(280 nm): 38,940 cm^−1^M^−1^ for HEWL and 8640 cm^−1^M^−1^ for RNase A. The final values were an average of 5 replicas.

### 3.2. Differential Scanning Calorimetry

Calorimetric measurements in solution were carried out using both a N-DSC III (model CS-6300, Calorimetry Science Corporation, Lindon, UT, USA) equipped with capillary cells and with a micro DSC III (Setaram, Caluire, France) equipped with 1.0 mL hermetically closed cylindrical pans, at scan rate 0.5 °C/min in the temperature range from 20.0 to 95.0 °C. For all the experiments, a heating-cooling cycle was performed, followed by a second heating ramp. All the samples were degassed and filtered before the measurements, following the standard procedure [26]. A blank scan was performed by filling the sample and the reference cells with the buffer, in the same experimental conditions.

Data were analysed using the software nano-Analyze Data Analysis (version 3.11.0, TA Instruments, New Castle, DE, USA) and Theseus [47], following a previously reported procedure [30,48]. Briefly, the excess molar heat capacity *C*_p_^exc^(T) of the sample, namely the difference between the apparent heat capacity and the protein heat capacity in the native state, was recorded across the scanned temperature range. The denaturation enthalpy (Δ_d_*H*) was defined as the area underlying the recorded peak. Errors were evaluated based on at least three replicas. The fit attempts based on the denaturation thermodynamic models (see the Supplementary Materials) were accomplished using the nonlinear Levenberg–Marquardt method [49].

The samples were tested in protein solutions at a concentration of 3 mg/mL (ca 2 × 10^−4^ M) in acetate buffer (0.1 M, pH 4.5), to which ca 30 μL of each metal complex was added at a concentration of 5 × 10^−2^ M in DMSO, with a complex/protein molar ratio of five. The amount of DMSO used to solubilize the Pt(II) complexes never exceed 2% *v*/*v*. The samples were incubated for 0, 24 or 48 h in a water-bath or a stove at 37 °C.

The effects of incubation at 37 °C on the thermal stability of the two model proteins used as reference were verified. The DSC thermograms showed slight differences as for the enthalpy values in the case of HEWL subjected to incubation. Indeed, despite both the peak profile and the denaturation temperature *T*_d_ were unaffected, a reduction of the enthalpy after 24 h and 48 h of incubation was observed, reflecting a loss of the protein native form (about 12% after 24 h and a further 9% after 48 h). Nonetheless, the profiles were perfectly superimposable after applying a correction that considered protein loss. For this reason, to correctly compare the mere effects of the compounds, the thermograms obtained for the interaction between HEWL and compounds **1**–**3** were all corrected with the same factor according to the incubation time. By contrast, the calorimetric profile of RNase A remains unaffected over incubation time, in agreement with the well-known good denaturation reversibility of RNase A reported in the literature [47,50].

### 3.3. UV-Vis Spectrophotometry

Both UV-2450 UV-Vis (Shimadzu, Chashan Town, Dongguan, China) and Lambda 35 UV-Vis (Perkin Elmer, Waltham, MA, USA) spectrophotometers were used. Both instruments are dual-beam ones; quartz cuvettes with an optical path of 1.0 cm were used.

To measure the stability of the metal complexes over time, the complex stock solution was diluted in acetate buffer (0.1 M, pH 4.5) to reach a concentration of 10^−5^ M. The absorption spectra were recorded at 25 °C.

To evaluate the interaction between the model proteins and each of the three Pt (II) complexes, differential spectrophotometric titrations were performed at 25 °C. For all titrations, first, a working solution of metal complex was obtained by adding 150 μL of 10^−3^ M stock solution in DMSO to 1.0 mL of 0.1 M acetate buffer at pH = 4.5. This was added to a solution of protein 2.0 × 10^−3^ M directly in the spectrophotometric cell. The additions were made by using a glass syringe connected to a micrometric screw (Mitutoyo–one turn equal to 8.2 μL, 1/50 of a turn is the minimum addition possible). Additions of titrant were done both in the cell containing the metal complex and in the reference cell so that the contribution of absorbance by the protein is zero (differential procedure). The experimental conditions require the presence of ca. 10% of DMSO in the sample, due to experimental needs and solubility limits of the complexes. Previous studies have shown that the protein structure is maintained at these levels of DMSO [51] and, due to the very limited dilution effect, the amount of DMSO in the sample stays constant all over the experiment. The absorption spectra are corrected for the dilution factor. Any titration was done at least in duplicate.

### 3.4. Mass Spectrometry

Stock solutions of HEWL and RNase A 10^−3^ M were prepared by dissolving the proteins in 2 × 10^−3^ M ammonium acetate solution (pH 6.8). Stock solutions 10^−2^ M of the investigated Pt compounds were prepared by dissolving the samples in DMSO.

For the ESI–MS experiments, aliquots of the protein stock solutions were mixed to aliquots of the Pt-based compounds stock solution in metal to protein ratio of 3:1 and diluted with ammonium acetate solution 2 × 10^−3^ M (pH 6.8) to a protein concentration of 10^−4^ M (in these conditions the final percentage of DMSO was 3%). The mixtures were incubated at 37 °C up to 48 h.

After 24 and 48 h of incubation time, all solutions were sampled and diluted to a final protein concentration of 10^−7^ M using ammonium acetate solution 2 × 10^−3^ M, pH 6.8 and added with 0.1% *v/v* of formic acid just before the infusion in the mass spectrometer.

The ESI mass spectra were acquired using a TripleTOF^®^ 5600^+^ high-resolution mass spectrometer (Sciex, Framingham, MA, USA), equipped with a DuoSpray^®^ interface operating with an ESI probe. All the ESI mass spectra were acquired through a direct infusion at 5 μL min^−1^ flow rate. The general ESI source parameters optimized for HEWL and RNase A were as follows: positive polarity; ionspray voltage floating 5500 V, temperature (TEM) 25 °C, ion source gas 1 (GS1) 45 L min^−1^; ion source gas 2 (GS2) 0; curtain gas (CUR) 20 L min^−1^, declustering potential (DP) 150 V, collision energy (CE) 10 V, acquisition range 1000–2600 *m*/*z*.

For the acquisition, the Analyst TF 1.7.1 software (Sciex, Framingham, MA, USA) was used and deconvolved spectra were obtained using the Bio Tool Kit v2.2 incorporated in the software PeakView™ v.2.2 (Sciex).

## 4. Conclusions

The joint use of different techniques permitted us to observe with a multi-technique approach the interaction between two model proteins (HEWL and RNase A) and three Pt(II) complexes, namely [PtCl(4′-phenyl-2,2′:6′,2″-terpyridine)](CF_3_SO_3_) (**1**), [PtI(4′-phenyl-2,2′:6′,2″-terpyridine)](CF_3_SO_3_) (**2**) and [PtCl(1,3-di(2-pyridyl)benzene)] (**3**), all potential cisplatin substitutes.

UV-Vis titrations permitted to detect the binding in short times of **1** and **2** complexes to HEWL (with a 1:1 stoichiometry) and to calculate apparent constant values of ca 10^4^ M^−1^. The significant spectral changes observed indicate that the aromatic part of the ligand is involved in the binding. Besides, the entropic destabilization and the peak asymmetry in the HEWL-complexes solutions observed by calorimetry suggested the occurring of non-specific interactions between the biosubstrate and the complexes. We hypothesize a statistical distribution of several populations in which the protein differently coordinates the metal complexes. Moreover, mass spectrometry permitted to exclude the covalent binding mode, suggested by the presence in HEWL + **1** of a small quantity of adduct complex-protein not stable in time. On the whole, the picture which comes out for complexes **1** and **2** is a binding mode mainly of electrostatic nature with the dicationic metal fragment and some negatively-charged amino acid residues, e.g., the aspartic acid. In addition to the electrostatic contribution, we believe that weak π-stacking interactions could play a role, as found by UV-Vis titrations.

We observed that steric and electronic differences of the ligands drive the nature of the interactions. The differences obtained for complexes **1** and **2** might be ascribable to the different reactivity of the labile ligand (Cl vs. I), and phenomenological evidence indicates that these compounds interact with the protein in peculiar manners. On the other hand, complex **3** (neutral) is less reactive and probably interacts with the protein only via weak hydrophobic interactions. Anyway, these interactions inhibit the substitution of the labile chloride ligand, as observed by UV-Vis spectroscopy.

The evaluation of the interactions between the three complexes and RNase A provided a different picture, as the complexes showed a stronger affinity with respect to the one observed with HEWL. Calorimetric measurements on protein-complex solutions showed kinetic effects, suggesting the occurrence of partial complex-induced protein denaturation during the incubation time. Besides, the appearance of a shoulder in the thermograms highlight the statistical distribution of multiple protein-complex species. The spectrophotometric analysis of the binding is made difficult complex phenomena that finally lead to precipitation. This finding may be related to the higher exposure of RNase A active sites, which enhances the possibility of aggregation phenomena. On longer time ranges, this accessibility turns into the ability of the metal complex to be placed in a position that enables covalent bond formation. This is enlightened by the mass spectra, which show that for RNase A + **2** the formation of 1:1 and 1:2 adducts after the labile ligand loss, whose abundance increase over time. Once again, complex **3** appears to be weakly reactive also with the RNase A.

This study provides information on the binding details of Pt(II)-terpy metal complexes to small proteins and on structure-reactivity relationships due to the ligands coordinated to the metal centre. It also evidences that the same Pt(II)-terpy metal complex may bind in a very different way to two apparently similar biosubstrates. Further studies, with the help of different techniques, will be needed to unravel selectivity issues of protein binding for this class of metal complexes.

## Figures and Tables

**Table 1:** Thermodynamic parameters of the unfolding process obtained for HEWL and RNase A in acetate buffer (0.1 M, pH = 4.5) alone and in presence of samples **1**–**3**, after 24/48 h of incubation at 37.0 °C.

Sample ^1^	Incub. (h)	*T_max_* (°C) ^2^	Δ_d_H (kJ mol^−1^) ^3^	Sample ^4^	Incub. (h)	*T_max_* (°C) ^2^	Δ_d_H (kJ mol^−1^) ^3^
HEWL	0	76.3	550	RNase A	0	59.4	440
HEWL + 1	24	71.8	515	RNase A + 1	24	56.4	220 *
	48	71.8	510				
HEWL + 2	24	71.8	550	RNase A + 2	24	51.8	420 *
	48	71.8	550				
HEWL + 3	24	75.5	550	RNase A + 3	24	57.6	425 *
	48	75.5	550				

^1^ Recorded by micro-DSC. ^2^ Denaturation temperature, the values are reported with an error bar of ± 0.2 °C. ^3^ Denaturation enthalpy, the values are reported with an RSD of 5%. ^4^ Recorded by nano-DSC. * These enthalpies are only apparent values because of kinetic effects.

**Table 2:** Binding constant parameters for the HEWL/Pt(II)-complex systems analysed in this work. Acetate buffer 0.1 M, pH = 4.5, 25 °C.

Titration	K (M^−1^) ^1^	K′ (M^−1^) ^2^	n ^2^
HEWL + **1**	(1.7 ± 0.2) × 10^4^	(1.5 ± 0.8) × 10^4^	0.91 ± 0.05
HEWL + **2**	(1.2 ± 0.4) × 10^4^	(1.2 ± 0.5) × 10^4^	1.00 ± 0.04
HEWL + **3**	(4 ± 2) × 10^2^	-	≅ 1.0

^1^ Obtained according to Equation (2). ^2^ Obtained according to Equation (3).

## Data Availability

The raw data will be made available upon request.

## Supplementary Materials

The following are available online. Figure S1: UV-Vis absorption spectra of the complexes **1** and **2** over time. Figure S2: Solutions of complex **3** at concentration 2.0 × 10^−4^ M in acetate buffer at time zero (**a**) and after one hour from solutions’ preparation (**b**); UV-Vis absorption spectra of the complex **3** in acetate buffer (**c**) and water (**d**) recorded over time. Figure S3: Thermograms for the proteins HEWL (**a**) and RNase A (**b**) recorded by micro-DSC and n-DSC. Figure S4: Micro-DSC thermograms of HEWL+ complex **1** (**a**), **2** (**b**) and **3** (**c**) after 24 h (green solid line) and 48 h (red dashed lines) of incubation in at 37.0 °C in 0.1 M acetate buffer at pH 4.5. Figure S5: Examples of UV-Vis titrations of the platinum complex **1** in the presence of increasing amounts of the HEWL protein and relevant data analysis. Figure S6: Examples of UV-Vis titrations of the platinum complex **2** in the presence of increasing amounts of the HEWL protein and relevant data analysis. Figure S7: Examples of UV-Vis titrations of the platinum complex **3** in the presence of increasing amounts of the HEWL protein and relevant data analysis. Figure S8: Absorption spectra of the platinum complex **2** in the presence of increasing amounts of the RNAse A protein (from blue to red) (**a**) and relevant binding isotherm at 410 nm (**b**). Relevant data analysis is also shown as panels (**c**) and (**d**). Figure S9: Deconvoluted ESI-Q-TOF mass spectra of HEWL incubated for 48 h at 37 °C with (**a** complex **1**, (**b**) **2** and (**c**) **3** in ammonium acetate solution. Figure S10: Deconvoluted ESI-Q-TOF mass spectra of HEWL incubated for 24 h at 37 °C with (**a**) complex **2** and (**b**) **3** in ammonium acetate solution. Figure S11: Deconvoluted ESI-Q-TOF mass spectra of RNase A incubated for 24 h at 37 °C with (**a**) complex **1**, (**b**) **2** and (**c**) **3** in ammonium acetate solution. Figure S12: Deconvoluted ESI-Q-TOF mass spectra of RNase A incubated for 48 h at 37 °C with (**a**) complex **1**, and (**b**) **3** in ammonium acetate solution.

## Sample Availability

Samples will made available upon request.
