# Supplementary material for: Thermodynamic Evaluation of the Interactions between Anticancer Pt(II) Complexes and Model Proteins
Source: Molecules. 2021 Apr 19;26(8):2376. doi: 10.3390/molecules26082376 (PMC8072931; doi:10.3390/molecules26082376)
Supplement: Supplementary file 1 [file molecules-26-02376-s001.zip › molecules-1153619-supplementary.pdf]

SUPPLEMENTARY MATERIALS

Thermodynamic Evaluation of Interactions between Anticancer Pt(II) Complexes and Model Proteins

Chiara Pelosi [1], Francesca Saitta [2], Caterina Zerino [1], Giovanni Canil [1], Tarita Biver [3,1], Alessandro Pratesi [1], Celia Duce [1], Dimitrios Fessas [2], Chiara Gabbiani [1,*] and Maria Rosaria Tiné [1,*]

[1] Department of Chemistry and Industrial Chemistry, University of Pisa, Via G. Moruzzi 13, 56124 Pisa, Italy; chiara.pelosi@dcci.unipi.it (C.P.); zerinocaterina@gmail.com (C.Z.); giocanil@gmail.com (G.C.); tarita.biver@unipi.it (T.B.); alessandro.pratesi@unipi.it (A.P.); celia.duce@unipi.it (C.D.)

[2] Dipartimento di Scienze per gli Alimenti, la Nutrizione e l'Ambiente, DeFENS, Università degli Studi di Milano, Via Celoria 2, 20133, Milano, Italy; francesca.saitta@unimi.it (F.S.); dimitrios.fessas@unimi.it (D.F.)

[3] Department of Pharmacy, University of Pisa, Via Bonanno Pisano 6, 56126 Pisa, Italy

* Correspondence: chiara.gabbiani@unipi.it (C.G.); mariarosaria.tine@unipi.it (M.R.T.); Tel.: +3905029225 (C.G.); +3905029268 (M.R.T.)

TABLE OF CONTENTS

PART 1: UV-VIS SPECTROPHOTOMETRY OF COMPLEX

PART 2: DIFFERENTIAL SCANNING CALORIMETRY

PART 3: UV-VIS TRITRATION

PART 4: MASS SPECTROMETRY

PART 1: UV-VIS SPECTROPHOTOMETRY OF COMPLEX

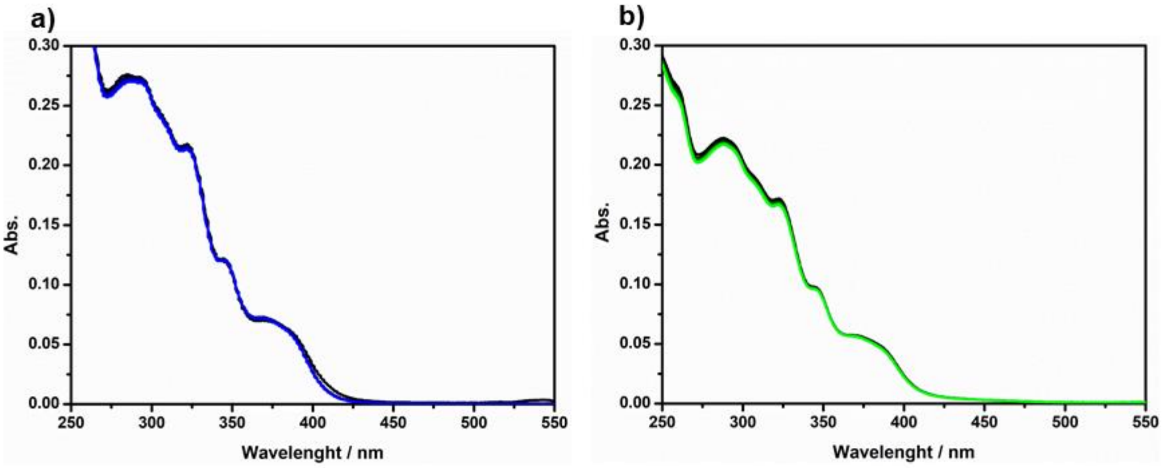

Figure S1. *UV-Vis absorption spectra of the complex* ***1*** *(a) and* ***2*** *(b) at concentration 1.0 x 10^{-5} M in 0.1 M acetate buffer at pH = 4.5, recorded over time. The spectra were recorded every hour from 0 to 24 hours. The colour gradation indicates the absorption spectra recorded at time zero (darkest line) up to 24 hours (lighter line).*

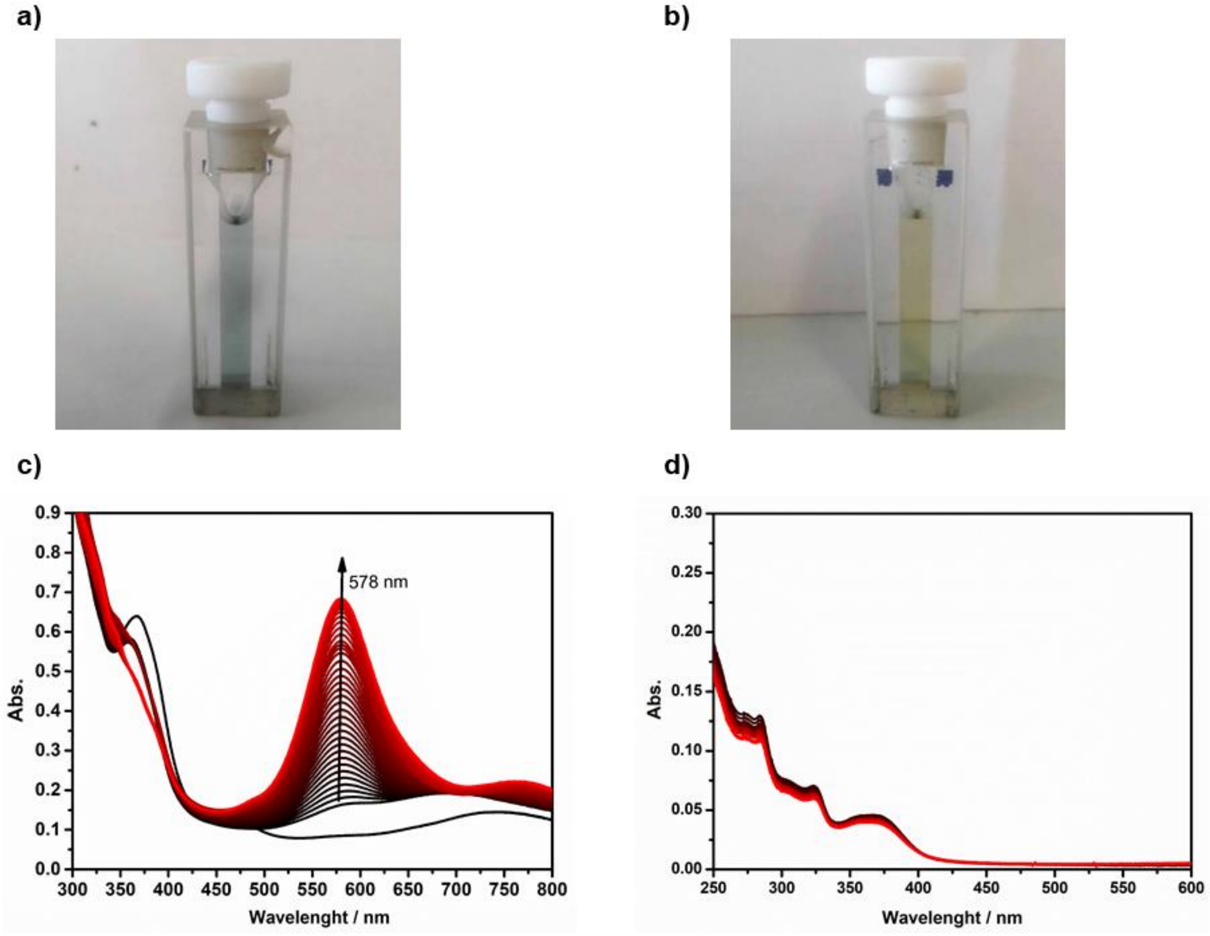

Figure S2. *Solutions of complex* ***3*** *at concentration 2.0 x 10^{-4} M in acetate buffer 0.1 M pH = 4.5 at time zero (a) and after one hour from solutions' preparation (b). UV-Vis absorption spectra of the complex* ***3*** *at concentration 1.4 x 10^{-4} M in 0.1 M acetate buffer at pH = 4.5 (c) and UV-Vis absorption spectra of the complex* ***3*** *at concentration 1 x 10^{-5} M in 0.1 M acetate buffer at pH = 4.5 in water (d) recorded over time. The spectra were recorded every hour from 0 to 40 hours in case of (c) and from 0 to 17 hours in case of (d). The colour gradation indicates the absorption spectra recorded at time zero (darkest line) up to 40 (or 17) hours (lighter line).*

PART 2: DIFFERENTIAL SCANNING CALORIMETRY

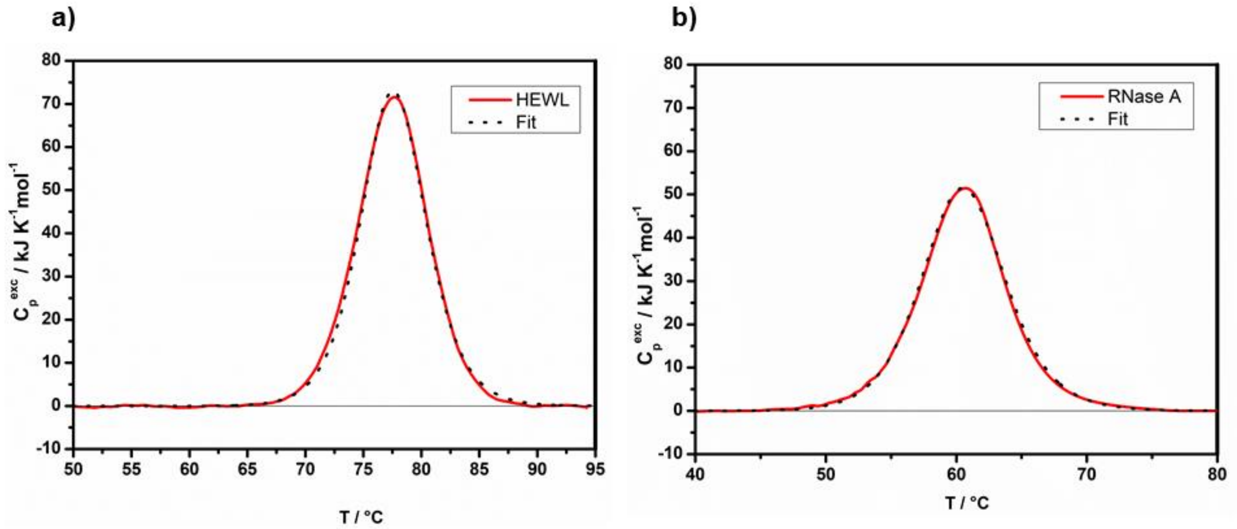

Figure S3. *Thermograms for the proteins HEWL (a) and RNase A (b) recorded by micro-DSC and n-DSC, respectively, in 0.1 M acetate buffer at pH 4.5 (black dotted curves). The red traces are the respective theoretical curves calculated according to a single-step denaturation model.*

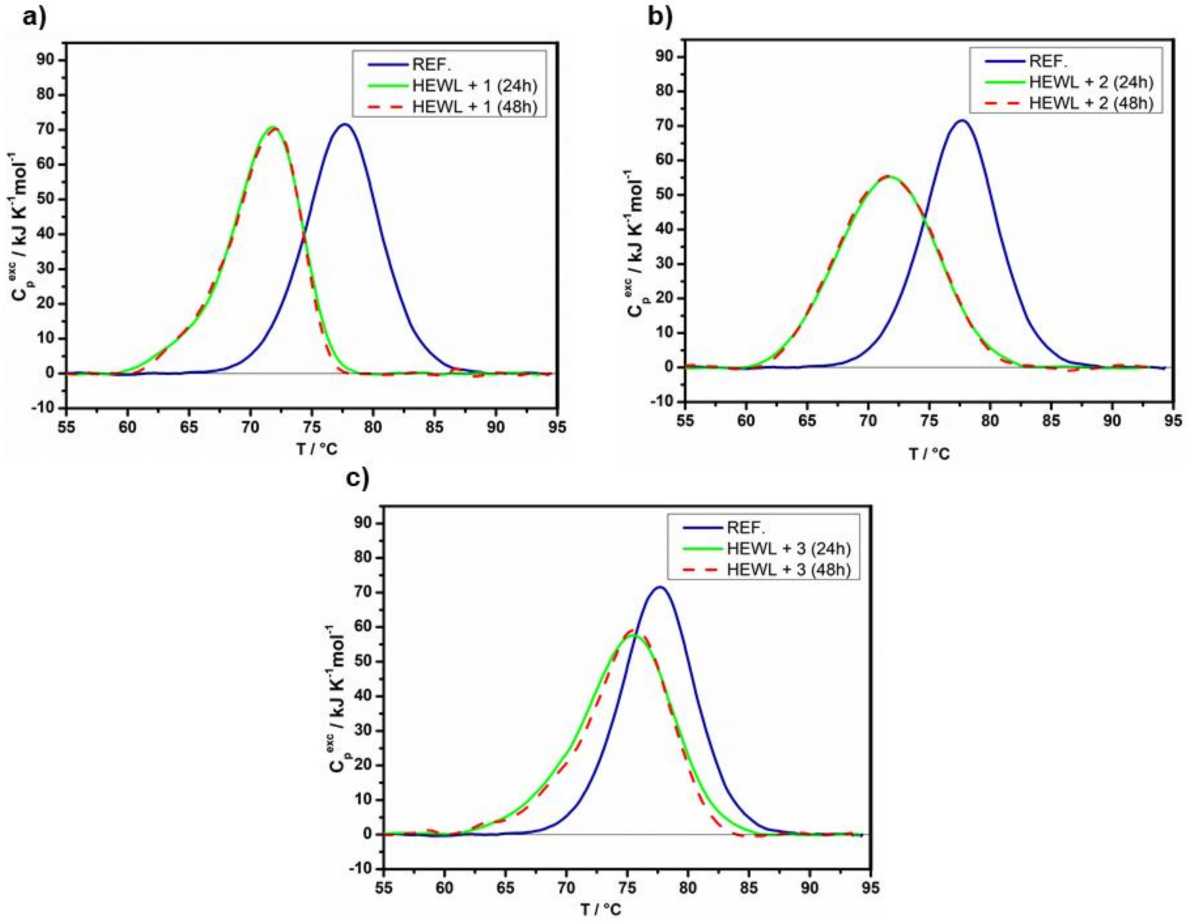

Figure S4. *Micro-DSC thermograms of HEWL+ complex* ***1*** *(a),* ***2*** *(b) and* ***3*** *(c) after 24 hours (green solid line) and 48 hours (red dashed lines) of incubation in at 37.0°C in 0.1 M acetate buffer at pH 4.5. We observe that the thermodynamic stability of the protein with the respective complex is not significantly affected from 24h to 48h incubation time (see Materials and Methods section in the main text).*

PART 3: UV-VIS TRITRATIONS

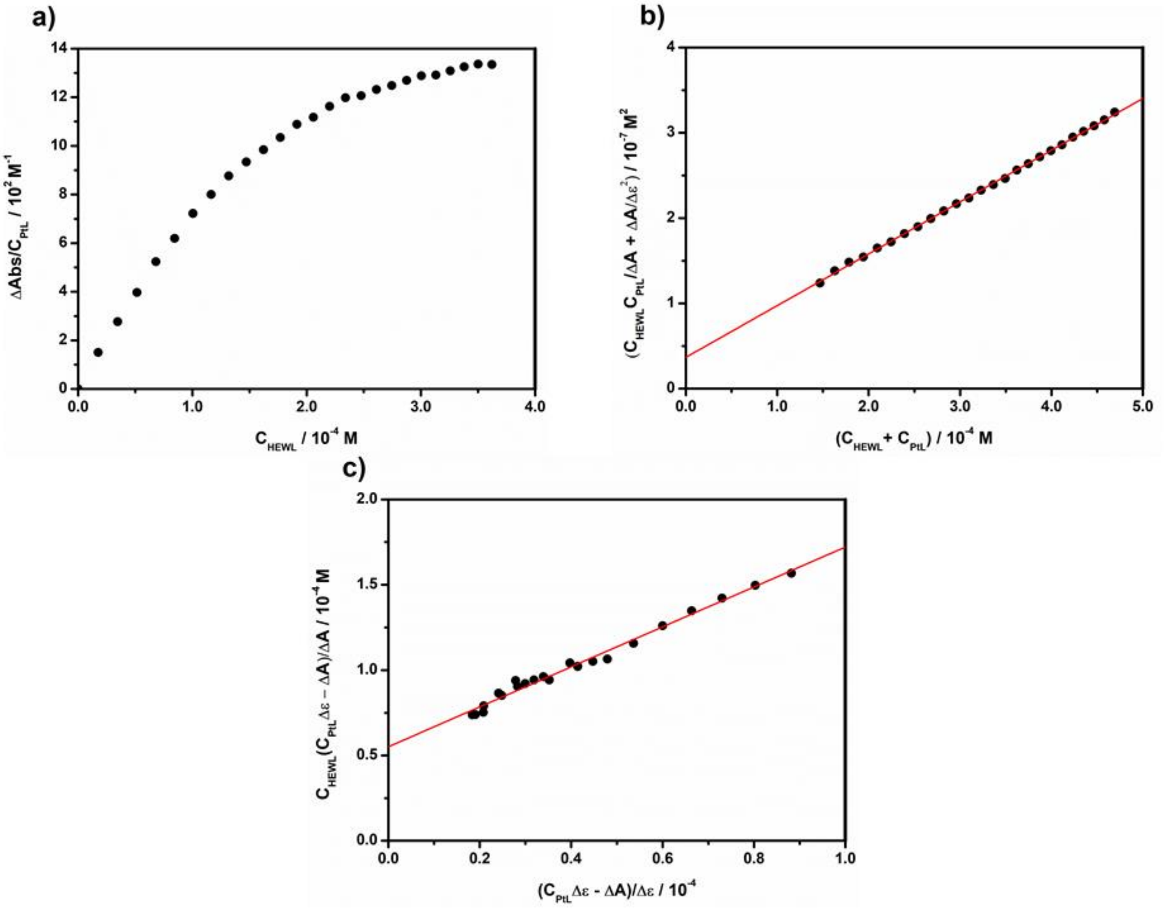

Figure S5. *Examples of UV-Vis titrations of the platinum complex* ***1*** *in the presence of increasing amounts of the HEWL protein and of relevant data analysis: (a) binding isotherm at 410 nm, (b) data analysis according to equation (2), (c) data analysis according to equation (3);* $C_{PtL} = 1.30\times10^{-4}$ *M;* C_{HEWL} *from 0 to* 3.57×10^{-4} *M; acetate buffer 0.1 M, pH = 4.5, 25°C.*

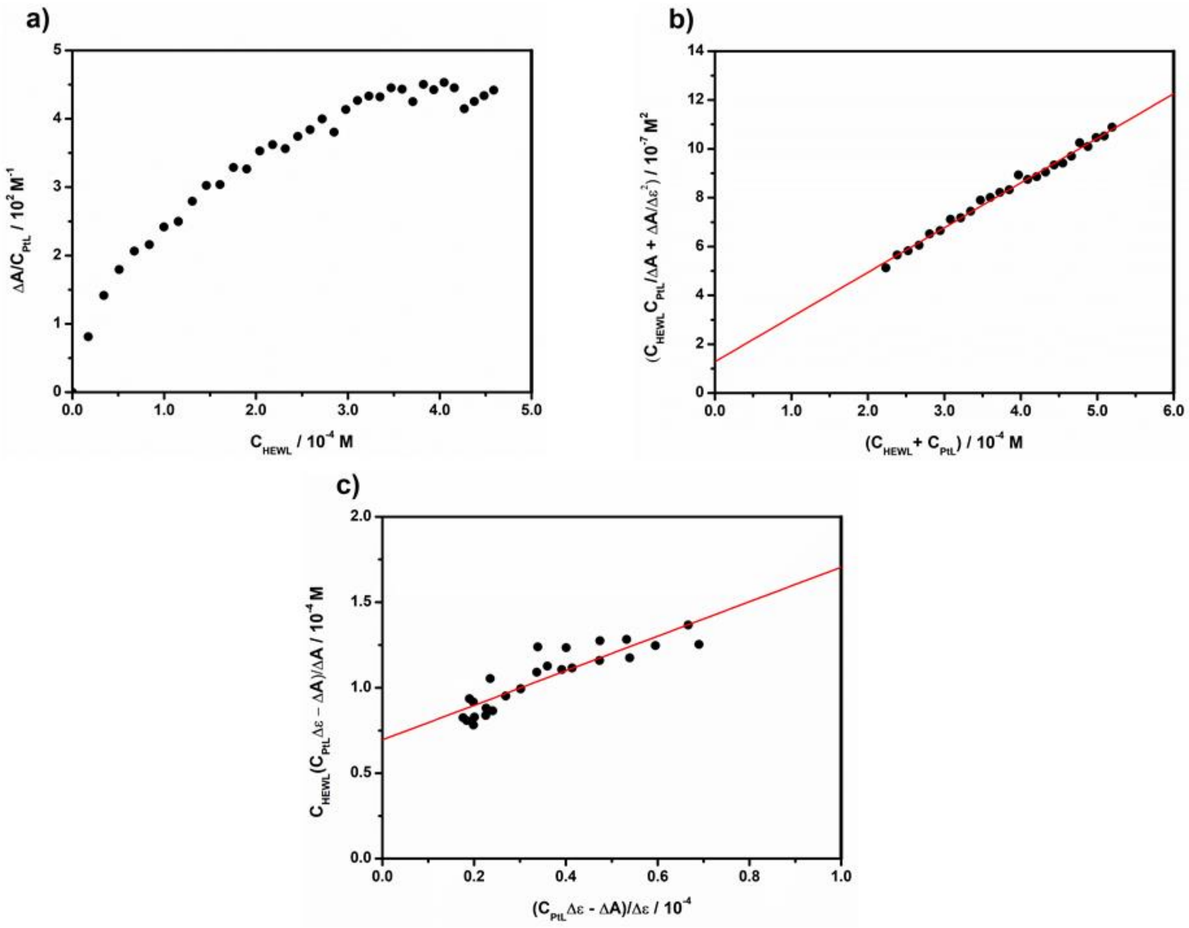

Figure S6. *Examples of UV-Vis titrations of the platinum complex* ***2*** *in the presence of increasing amounts of the HEWL protein and of relevant data analysis: (a) binding isotherm at 395 nm, (b) data analysis according to equation (2), (c) data analysis according to equation (3);* $C_{PtL} = 1.30\times10^{-4}$ *M;* C_{HEWL} *from 0 to* 4.59×10^{-4} *M; acetate buffer 0.1 M, pH = 4.5, 25°C.*

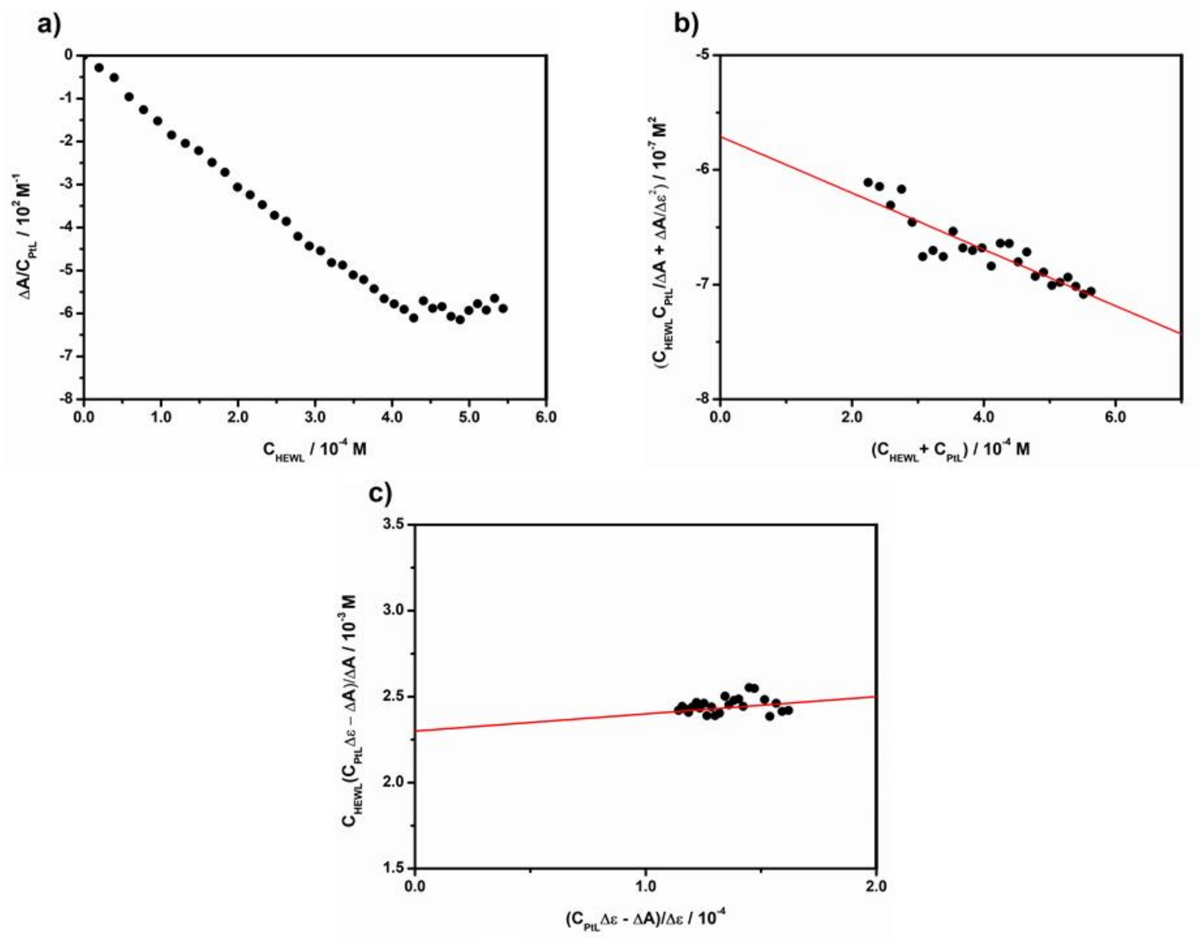

Figure S7. *Examples of UV-Vis titrations of the platinum complex* ***3*** *in the presence of increasing amounts of the HEWL protein and of relevant data analysis: (a) binding isotherm at 700 nm, (b) data analysis according to equation (2), (c) data analysis according to equation (3); C_{PtL} = 1.71×10^{-4} M; C_{HEWL} from 0 to 5.44×10^{-4} M (but data above 4×10^{-4} M suffer precipitation and cannot be used in the analyses); acetate buffer 0.1 M, pH = 4.5, 25°C.*

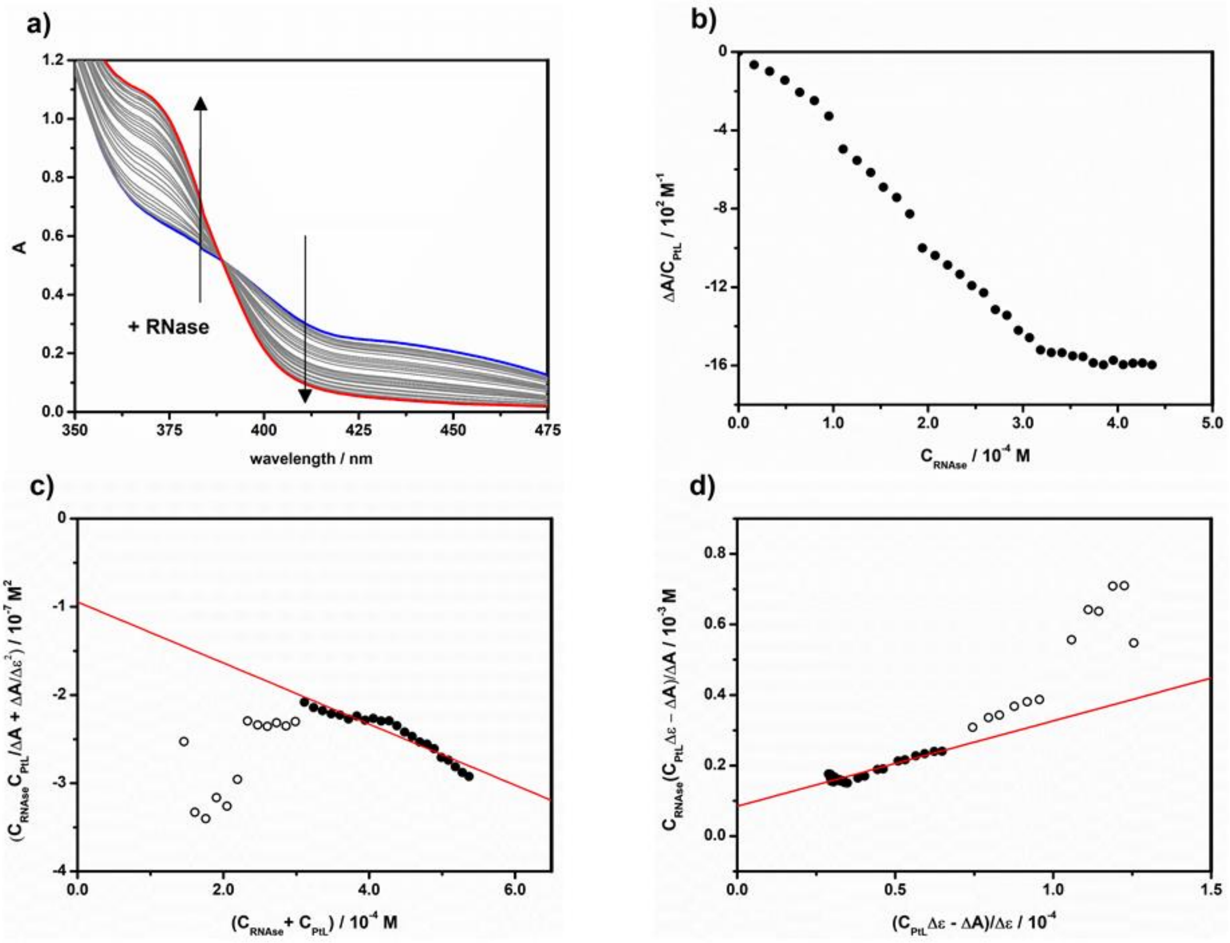

Figure S8. *Absorption spectra of the platinum complex* ***2*** *in the presence of increasing amounts of the RNase A protein (from blue to red) (a) and relevant binding isotherm at 410 nm (b). Relevant data analysis according to eq. (2) and (3) are also shown as panels (c) and (d) respectively;* C_{PtL} *= 1.30×10*$^{-4}$ *M;* C_{RNase} *from 0 to 4.36×10*$^{-4}$ *M; acetate buffer 0.1 M, pH = 4.5, 25°C.*

PART 4: MASS SPECTROMETRY

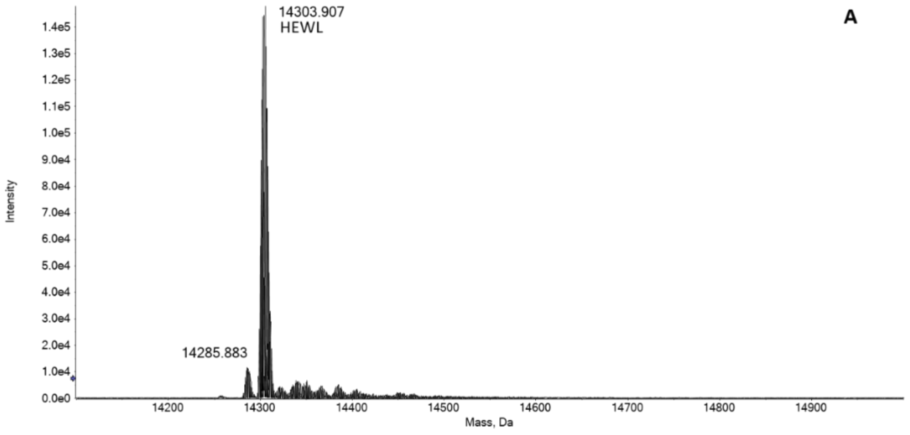

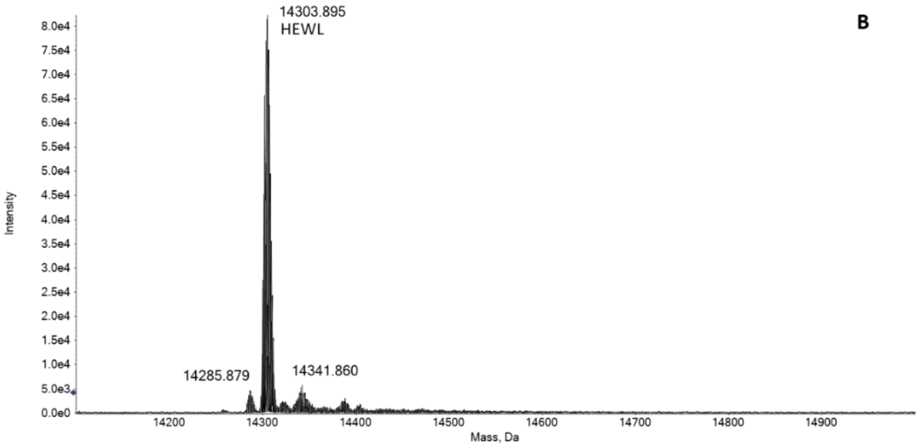

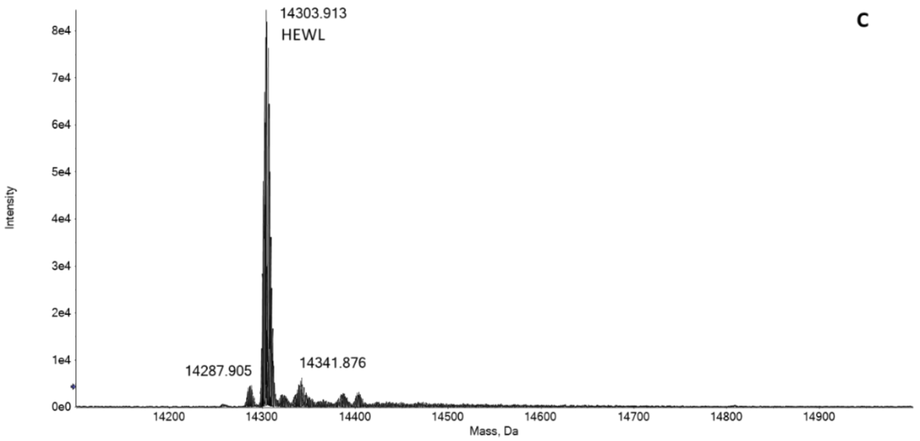

Figure S9. *Deconvoluted ESI-Q-TOF mass spectra of HEWL 10^{-7} M incubated for 48 h at 37 °C with (A) complex **1**, (B) **2** and (C) **3** (1:3 protein/complex ratio) in ammonium acetate solution 2×10^{-3} M (pH 6.8).*

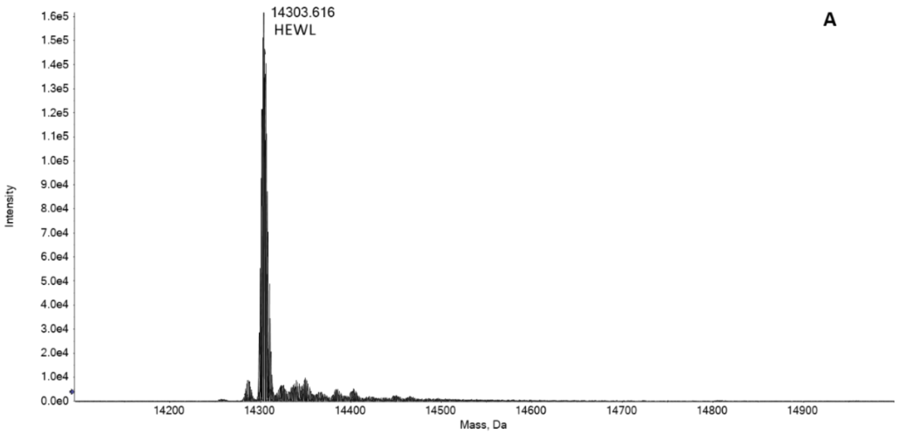

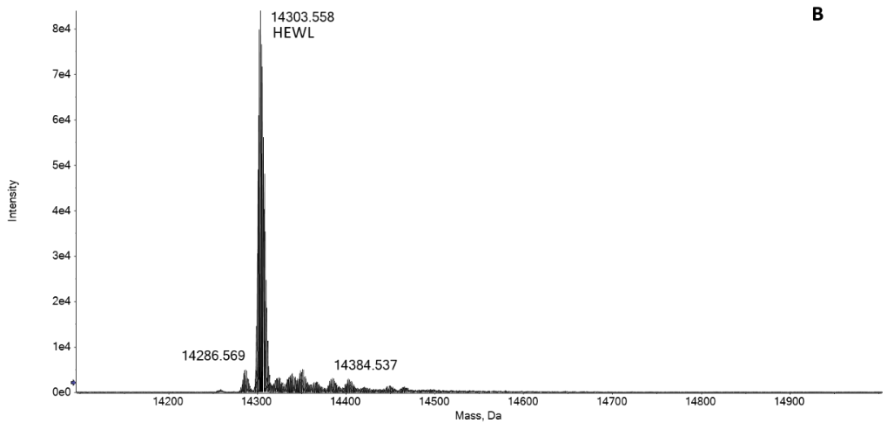

Figure S10. *Deconvoluted ESI-Q-TOF mass spectra of HEWL 10^{-7} M incubated for 24 h at 37 °C with (A) complex* ***2*** *and (B)* ***3*** *(1:3 protein/complex ratio) in ammonium acetate solution 2×10^{-3} M (pH 6.8).*

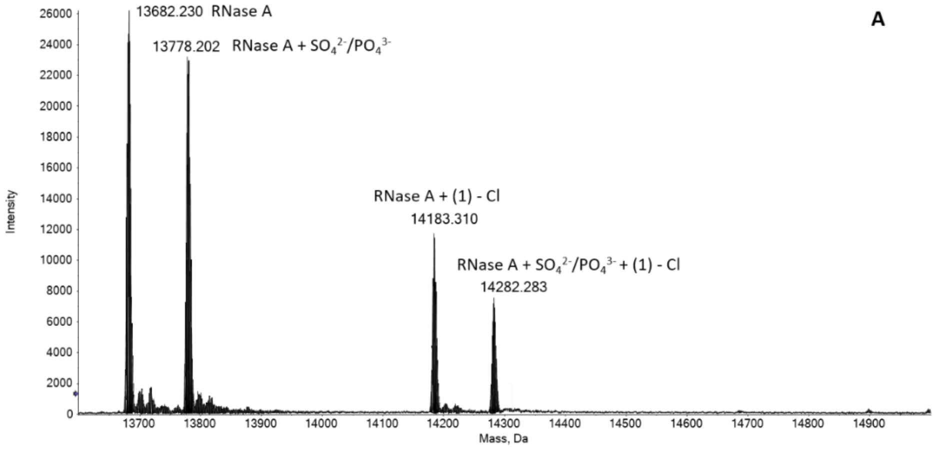

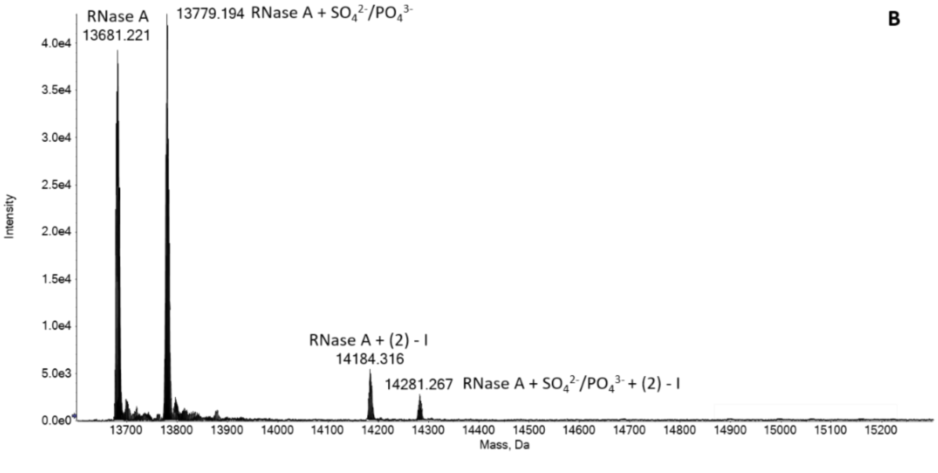

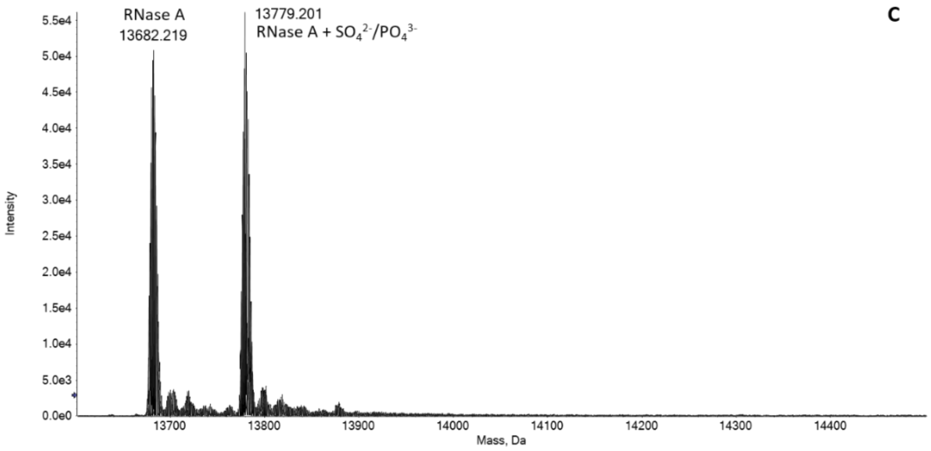

Figure S11. *Deconvoluted ESI-Q-TOF mass spectra of RNase A 10^{-7} M incubated for 24 h at 37 °C with (A) complex* ***1****, (B)* ***2*** *and (C)* ***3*** *(1:3 protein/complex ratio) in ammonium acetate solution 2 × 10^{-3} M (pH 6.8).*

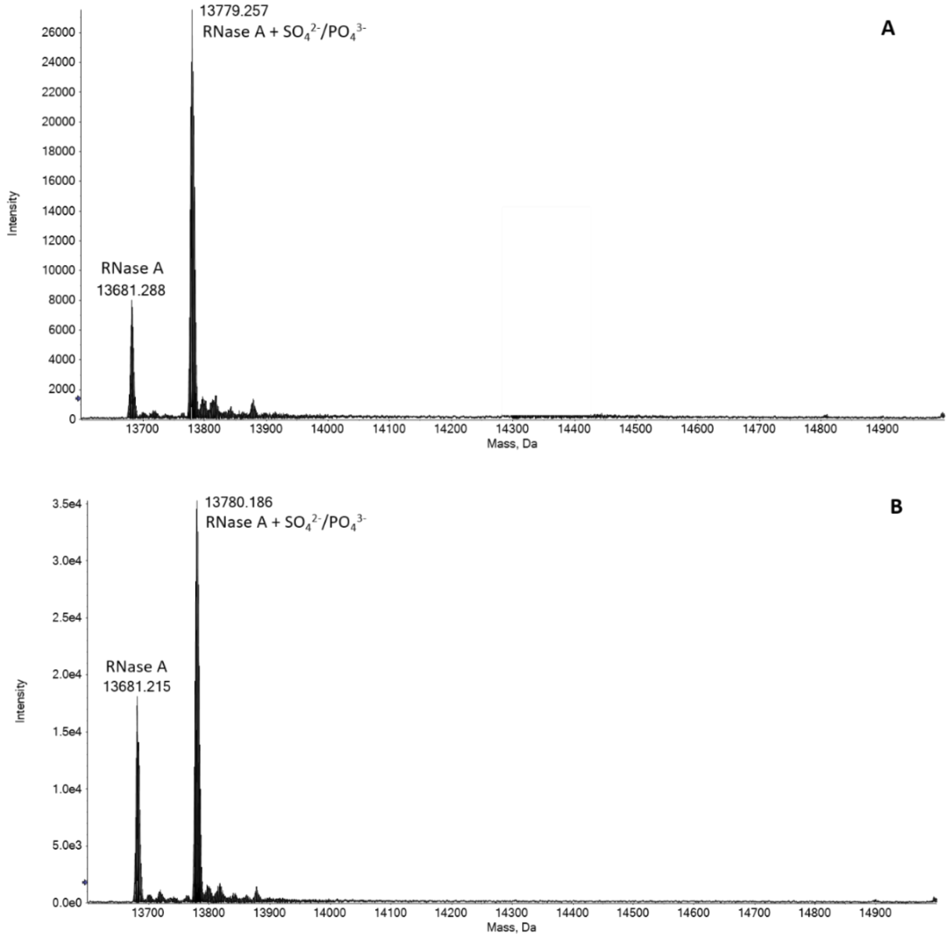

Figure S12. *Deconvoluted ESI-Q-TOF mass spectra of RNase A 10^{-7} M incubated for 48 h at 37 °C with (A) complex* ***1****, and (B)* ***3*** *(1:3 protein/complex ratio) in ammonium acetate solution 2 × 10^{-3} M (pH 6.8).*
